# Multimodal imaging of foveoschisis and macular pseudohole associated with gyrate atrophy: a family report

**DOI:** 10.1186/s12886-018-0755-9

**Published:** 2018-04-12

**Authors:** Imène Zhioua Braham, Imen Ammous, Rim Maalej, Majdi Boukari, Ilhem Mili Boussen, Khalil Errais, Raja Zhioua

**Affiliations:** Department of Ophthalmology, Charles Nicolle University Hospital, Faculty of medicine of Tunis, University of Tunis-El Manar, 1007 Tunis, Tunisia

**Keywords:** Gyrate atrophy, Foveoschisis, Macular pseudohole, Optical coherence tomography angiography

## Abstract

**Background:**

To report the results of multimodal imaging of a biochemically confirmed case of a family with gyrate atrophy (GA) associated with foveoschisis and macular pseudohole.

**Case presentation:**

Two sisters presented to us with progressive bilateral decreased vision. The 26-year old sister had a best corrected visual acuity (BCVA) of 20/32 in the right eye (RE) and 20/100 in the left eye (LE). Dilated fundus examination revealed multiple bilateral chorioretinal atrophy areas in the midperipheral and peripheral retina. Fluorescein angiography did not show any leak in the macular area. Swept-source optical coherence tomography (SS-OCT) showed increased central macular thickness in both eyes with foveoschisis. Optical coherence tomography angiography (OCTA) showed petaloid non-reflective areas and some perifoveal microvascular alterations similar to telangiectasias in the deep capillary complex. The 30-year-old sister had a BCVA of 20/20 in the RE and 20/32 in the LE. SS-OCT was normal in the RE and demonstrated a macular pseudohole with a fine epiretinal membrane in the LE. The persistent retinal tissue at the base of the pseudohole was disorganised. Blood tests showed hyperornithinemia in the 2 cases. Based on these observations, the patients were diagnosed with gyrate atrophy of the choroid and retina and were treated with a pyridoxine supplement and an arginine-restricted diet.

**Conclusions:**

Foveoschisis and macular pseudohole may be associated in GA, increasing the risk of rapid vision loss. OCTA is an interesting imaging tool that can help to better understand the pathophysiological mechanism of these macular involvements in GA.

## Background

Gyrate atrophy (GA) of choroid and retina is a rare disease with recessive autosomal transmission [1]. It is a metabolic disorder secondary to a congenital deficit in *ornithine-δ-aminotransferase (OAT)* causing hyperornithinemia. With an unknown mechanism, high levels of ornithine lead to a progressive chorioretinal atrophy [2, 3]. Patients with GA initially complain of night blindness and decreasing of peripheral vision typically in the second decade of life. Loss of central vision can occur in the fourth or the fifth decades.

Ophthalmological manifestations such as high myopia, marked astigmatism and early cataract formation are common [4]. Macular involvements can be due to macular edema, choroidal neovascularisation, macular hole and unfrequently foveoschisis and epiretinal membrane [5]. Two cases of foveoschisis and no case of macular pseudohole associated with GA, were reported [6, 7].

Here, we report the results of multimodal imaging of a biochemically confirmed case of a family with GA associated with foveoschisis and macular pseudohole. To our knowledge, this is the first report of GA imaged by optical coherence tomography angiography (OCTA) in the indexed peer-reviewed literature.

## Case presentation

A 26 year-old and a 30-year-old Caucasian sisters presented to us with progressive bilateral decreased vision. There was no history of consanguineous marriage.

### Case 1

**Fig. 1:**
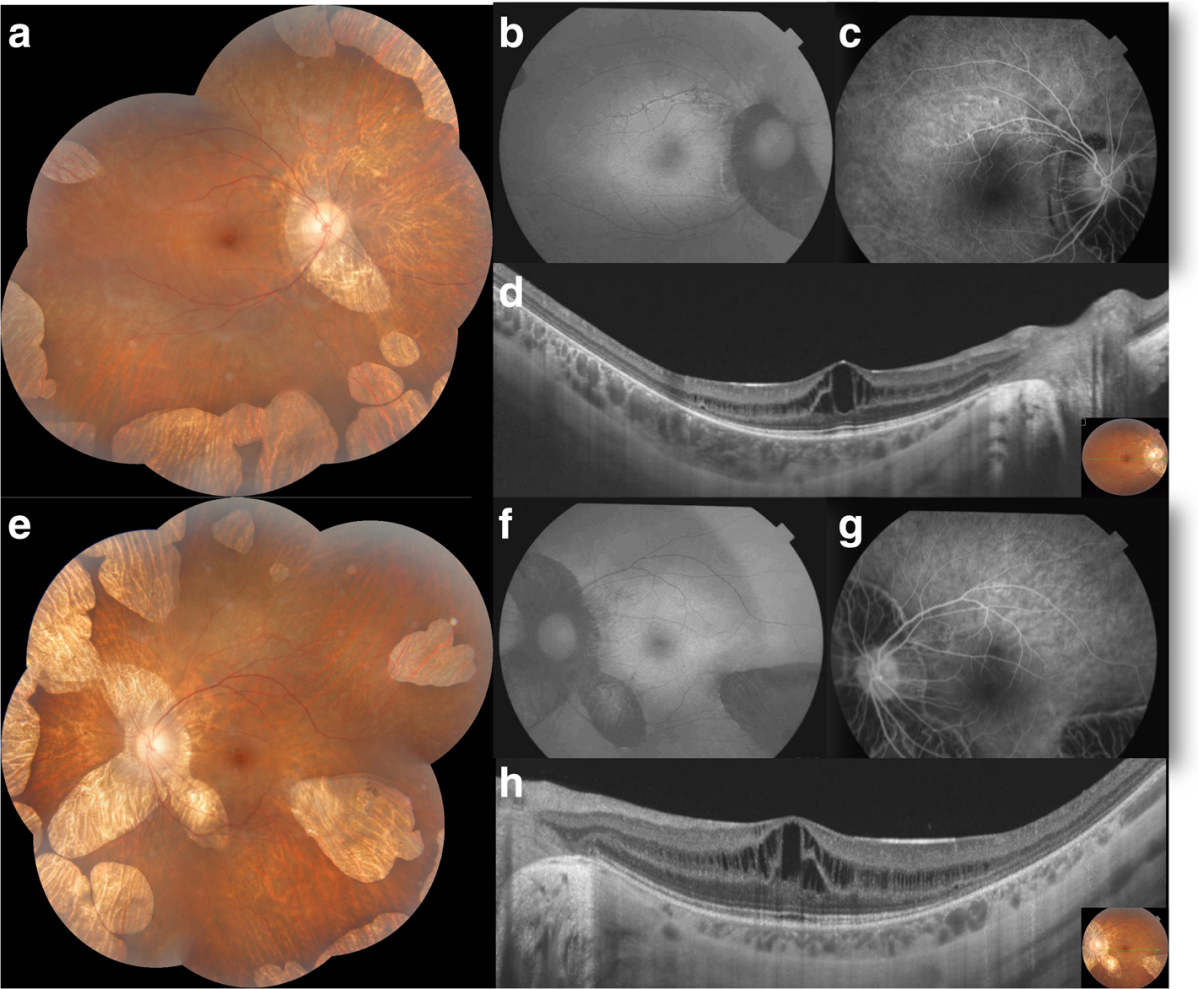
Case 1. Bilateral foveoschisis associated with gyrate atrophy. **a,e.** Composite colored fundus photography of both eyes showed multiple areas of well circumscribed chorioretinal atrophy in the midperipheral and peripheral zone. **b,f.** Fundus autofluorescence of both eyes revealed decreased autofluorescence in the areas of chorioretinal atrophy. **c,g.** Fluorescein angiography in the early phase (**c**) and in the late phase (**g**) didn’t reveal any leak in the macular area in both eyes. **d,h.** SS-OCT demonstrated a thickening of the macula with multiple hyporeflective spaces in the inner nuclear layer separated by vertical bridges suggesting foveoschisis in both eyes

Best corrected visual acuity (BCVA) was 20/32 in the right eye (RE) and 20/100 in the left eye (LE) with a refraction error of − 11 diopter (D) / -3,00 D × 90° and − 8,50 D/ -2,50 D × 5°, respectively. Biomicroscopic examination of the anterior segment was unremarkable. Dilated fundus examination revealed multiple bilateral, sharply defined and well circumscribed chorioretinal atrophy areas in the midperipheral and peripheral retina (Fig. 1a,e). Fundus autofluorescence showed decreased autofluorescence that correlated with chorioretinal atrophy areas (Fig. 1b,f). Fluorescein angiography did not show any leak in the macular area even in the late phase (Fig. 1c,g).

Swept-source optical coherence tomography (SS-OCT) showed increased central macular thickness at 398 μm in the RE and 355 μm in the LE. The presence of hyporeflective spaces extending from the outer plexiform layer to the inner plexiform layer, separated by multiple vertical bridges was suggestive of foveoschisis on both eyes (Fig. 1d,h).

**Fig. 2:**
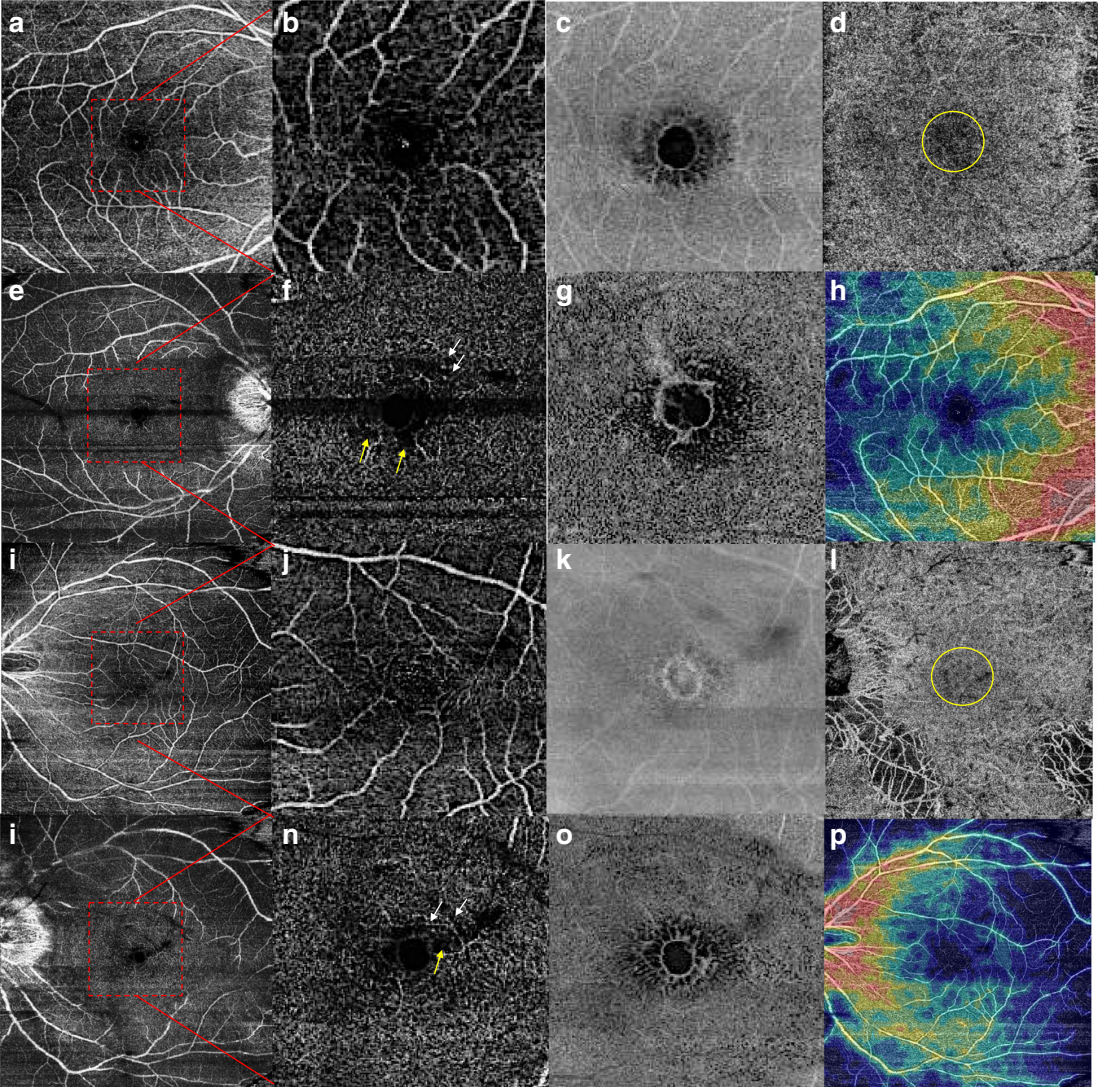
Case 1. Optical coherence tomography angiography and En-Face swept-source OCT in bilateral foveoschisis associated with gyrate atrophy. **a,b,i,j.** Superficial capillary plexus (9x9mm image) was normal in both eyes. The red square outlines the area of the 3x3mm magnified (**b,j**). **e,f,m,n.** Deep capillary plexus (**e,m:** 9x9mm, **f,n:** 3x3mm images) showed some perifoveal microvascular alterations similar to telangiectasias (white arrows) and petaloid non-reflective areas (yellow arrows). **c,g,k,o.** En-face images of superficial (**c,k**) and deep capillary plexus (**g,o**) of both eyes revealed a honeycomb pattern of the hyporeflective spaces better seen in the deep plexus. **d,i.** Choriocapillary layer in both eyes showed a central grey layer that could be due to a shadow effect (yellow circle). **h,p.** Density map of both eyes

The En-Face SS-OCT demonstrated the exact extent and anatomical configuration of the hyporeflective spaces, well seen in the inner layers with a honeycomb pattern (Fig. 2).

OCTA showed petaloid non-reflective areas in the macular zone located predominantly inside of the deep capillary complex. Besides, there was evidence of some perifoveal microvascular alterations similar to telangiectasias located in the deep capillary plexus. Analysis of the choriocapillaris revealed a central dark grey area that was not associated with any evident aspect of a vascular alteration but could be attributed to a decreased signal due to a shadow effect (Fig. 2).

The visual field showed peripheral alterations and electrophysiology revealed reduced photopic responses with no recordable scotopic response.

Clinical aspect and multimodal imaging identified gyrate atrophy of the choroid and retina. Neurological examination was normal. Elevated plasma ornithine test confirmed the diagnosis (723 nmol/ml, with the normal range being 50 to 100 nmol/ml). An arginine-restricted diet and B6 vitamin supplementation (at dose of 300 mg/day) were prescribed. After a six-month follow-up, no retinal changes were observed.

### Case 2

**Fig. 3:**
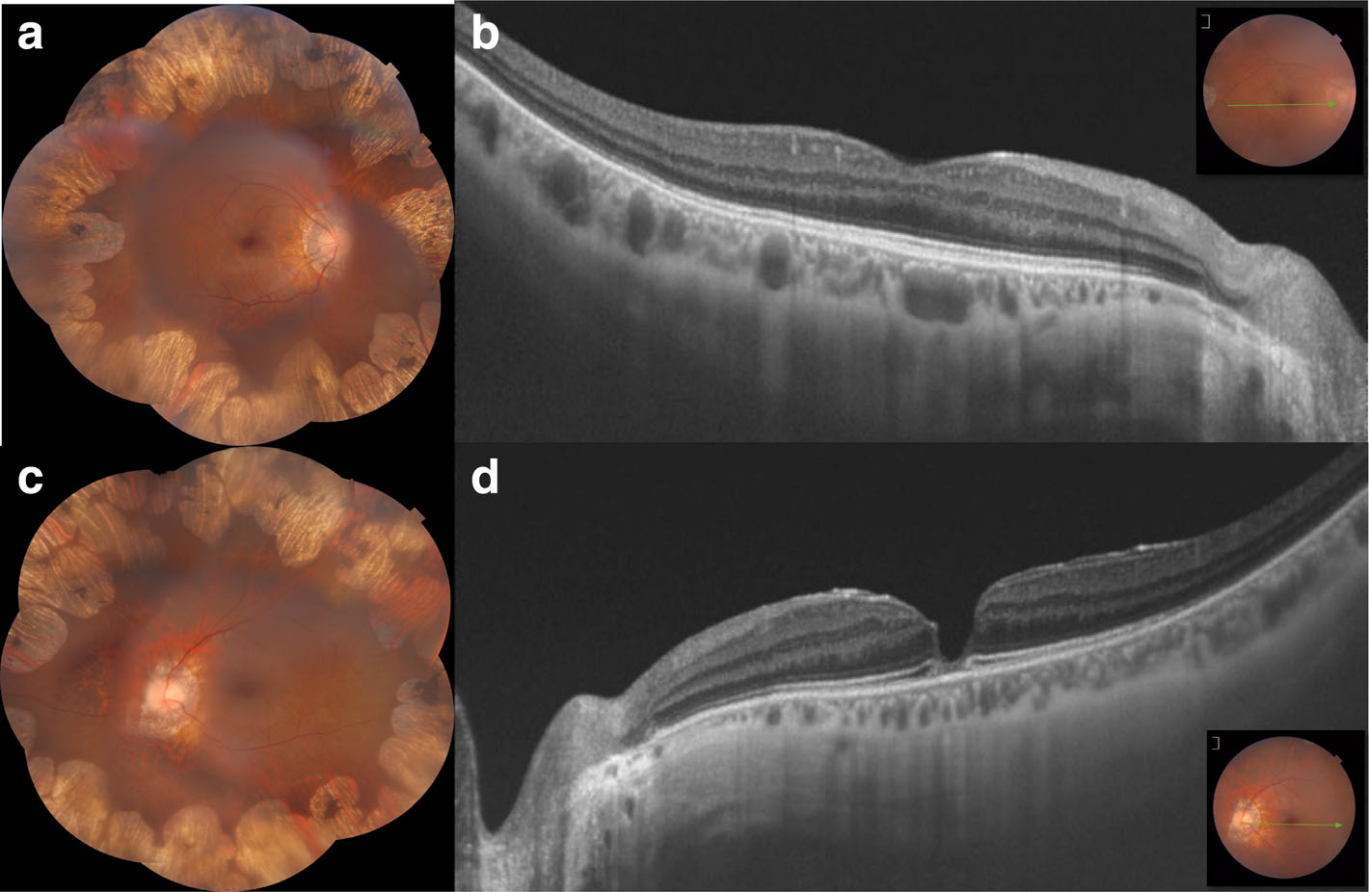
Case 2: Macular pseudohole associated with gyrate atrophy. **a,c.** Composite colored fundus photography of both eyes showed coalescent areas of peripheral chorioretinal atrophy sparing the posterior pole. **b,d.** SS-OCT was normal in the RE and revealed a macular pseudohole with a fine epiretinal membrane in the LE with a disorganised ellipsoid zone at the base of the pseudohole

The 30-year-old sister was pseudophakic in both eyes. BCVA was 20/20 in the RE and 20/32 in the LE with a refractive error of − 1,25 D / -0.75 D × 20° and − 0,75 D /− 1.25 D × 15°, respectively. Biomicroscopic examination of the anterior segment was unremarkable. Dilated fundus examination revealed multiple, coalescent areas of peripheral chorioretinal atrophy sparing the posterior pole in both eyes. SS-OCT was normal in the RE and demonstrated a macular pseudohole with a fine epiretinal membrane in the LE. The persistent retinal tissue at the base of the pseudohole was limited to a disorganised ellipsoid zone with thinning of the retina at the fovea (Fig. 3). The En-Face SS-OCT didn’t show any distorsion of the fovea nor retinal folds.

The visual field and electrophysiology showed the same alterations as in the first case. No systemic manifestations were observed. Diagnosis of GA was confirmed by elevated plasma ornithine test.

The clinical examination of the parents was within normal.

## Discussion

Gyrate atrophy is a recessive autosomic disease affecting the *OAT* whose deficiency causes elevation of ornithine. The initial lesion is located on the pigmentary epithelium causing atrophy of the photoreceptors [8, 9]. In GA, by the age of 10, gradual blurred vision starts, becomes severe in the fourth or fifth decade. Rare and fine hair, mental retardation associated with diffuse cerebral atrophy and muscle damage, are the most described systemic manifestations [10].

Described macular involvements in GA are pigment epithelial window defects, patches of chorioretinal atrophy, cystoid macular edema, epimacular membrane and choroidal neovascularization [4, 5, 11].

Foveoschisis is a rare association with GA. To the best of our knowledge, this is the third report of this association [6, 7]. There were no previous reports of OCTA in GA. In case 1, the aspect of intra retinal cysts on SS-OCT and the absence of leak at macula at the late phase of FA was suggesting foveoschisis rather than cystoid macular edema.

Foveoschisis corresponds to the splitting of the inner retinal layers at the macula. In high myopia, foveoschisis is probably related to vitreoretinal interface factors, to a progressive ectasia of the sclera and a relative resistance to a stretch of the inner retinal layers and the retinal vessels [12, 13]. In X-linked retinoschisis, mutations in the RS1 gene cause a loss of functional retinoschisin which disturbs the cellular organization of the retina and structure of the photoreceptor-bipolar synapse [14]. In GA, the pathogenesis of the foveoschisis is poorly understood. *OAT* activity is reported to be high in the retinal pigment epithelium which can explain that the initial lesion in GA is located on the pigmentary epithelium causing atrophy of the photoreceptors [8, 9]. So we can hypothesize that deficiency of essential products or toxicity of the accumulated excessive substrates, causing the chorioretinal degeneration in GA, may also disturb regulation of the fluid balance between the intracellular and extracellular environment. The extracellular fluid with cystic cavities causes disruption of the organized layers of the retina particularly within the photoreceptor and bipolar cell layers. However, pathogenesis of the foveoschisis in gyrate atrophy is probably multifactorial and can be related to vitreoretinal interface factors and to high myopia as in our patient [12]. Nevertheless, high myopia is not always involved, as Tekin et al. described a case of foveoschisis associated with GA in low myopia [7].

The OCTA showed in our case some perifoveal microvascular alterations located in the deep capillary plexus which can be either a cause or a consequence of the foveoschisis. Starga et al. found similar macular lesions in cases of X-linked retinoschisis [15].

Further studies with OCTA imaging need to be performed to better define the pathogenesis of foveoschisis in GA.

Epiretinal membrane is also a rare association with GA [16]. To the best of our knowledge, we report the first case of macular pseudohole imaged and published. Regarding its pathogenesis, several factors including aging, posterior vitreous detachment and genetic factors have been suggested to play a role in epiretinal membrane formation. In our case, the patient was young and macular pseudohole aspect was unusual as external retinal layers were disorganised and poorly individualized at the base of the pseudohole. This aspect must be distinguished from full-thickness macular hole, which has been also reported as a rare finding in GA [17].

In the treatment of GA, a low-protein diet, but sufficient for normal growth, is recommended. An arginine-free diet, if strictly obeyed, may decrease ornithine to normal levels but only 20% of patients are reported to tolerate this diet [18]. Oral B6 supplementation is administered in order to decrease serum ornithine level with a clinical heterogeneity in responsiveness [19]. Recently, Heller et al. reported for the first time an improvement of central macular edema with low protein intake and pyridoxine supplement [20]. However, treatment effect on foveoschisis is still unknown. In our patient, a longer follow-up period is needed to evaluate the long-term treatment effect.

## Conclusions

Association of GA with foveoschisis and macular pseudohole is rare but may increase the risk of rapid vision loss. The recent widespread clinical use of optical coherence tomography has better defined characteristics of macular abnormalities in GA. OCTA is an interesting imaging tool that can help to better understand the pathophysiological mechanism of macular involvements in GA.

## Abbreviations

BCVA: Best corrected visual acuity
D: Diopter
GA: Gyrate atrophy
LE: Left eye
OAT: Ornithine-δ-aminotransferase
OCTA: Optical coherence tomography angiography
RE: Right eye
SS-OCT: Swept-source optical coherence tomography
